# The Future of Point-of-Care Nucleic Acid Amplification Diagnostics after COVID-19: Time to Walk the Walk

**DOI:** 10.3390/ijms232214110

**Published:** 2022-11-15

**Authors:** Juan García-Bernalt Diego, Pedro Fernández-Soto, Antonio Muro

**Affiliations:** Infectious and Tropical Diseases Research Group (e-INTRO), Biomedical Research Institute of Salamanca-Research Centre for Tropical Diseases at the University of Salamanca (IBSAL-CIETUS), Faculty of Pharmacy, University of Salamanca, 37007 Salamanca, Spain

**Keywords:** SARS-CoV-2, RT-qPCR, isothermal amplification, point-of-care diagnostics, CRISPR, variants

## Abstract

Since the onset of the COVID-19 pandemic, over 610 million cases have been diagnosed and it has caused over 6.5 million deaths worldwide. The crisis has forced the scientific community to develop tools for disease control and management at a pace never seen before. The control of the pandemic heavily relies in the use of fast and accurate diagnostics, that allow testing at a large scale. The gold standard diagnosis of viral infections is the RT-qPCR. Although it provides consistent and reliable results, it is hampered by its limited throughput and technical requirements. Here, we discuss the main approaches to rapid and point-of-care diagnostics based on RT-qPCR and isothermal amplification diagnostics. We describe the main COVID-19 molecular diagnostic tests approved for self-testing at home or for point-of-care testing and compare the available options. We define the influence of specimen selection and processing, the clinical validation, result readout improvement strategies, the combination with CRISPR-based detection and the diagnostic challenge posed by SARS-CoV-2 variants for different isothermal amplification techniques, with a particular focus on LAMP and recombinase polymerase amplification (RPA). Finally, we try to shed light on the effect the improvement in molecular diagnostics during the COVID-19 pandemic could have in the future of other infectious diseases.

## 1. Introduction

COVID-19, caused by the *betacoronavirus* SARS-CoV-2, is the last pandemic humanity has suffered. However, it has been preceded by various viral outbreaks since the start of the 20th century. Beginning with the Spanish Flu in 1918, which infected a third of the population at the time and caused at least 50 million deaths [1], various pandemics, major viral epidemics and outbreaks have occurred. They include three more influenza pandemics, the Asian Flu in 1957, the Hong Kong Flu in 1968, and the H1N1 Influenza A pandemic in 2009, as well as numerous epidemics [2,3]. Besides influenza viruses, outbreaks from deadlier viruses, such as Zaire-Ebola virus, have been an increasing concern [4]. Already in the 21st century, other betacoronaviruses, specifically SARS-CoV and MERS-CoV, have caused alarming sprouts [5]. Consequently, the responses to previous viral outbreaks have informed the myriad of scientific approaches developed to stop the current one [6]; as the experience acquired and mistakes made throughout the response to the actual crisis should enlighten the preparation for future emergencies [7].

A key lesson drawn from the pandemic management and control strategies is that the most efficient way to prevent disease transmission is the identification of as many infected individuals as possible, regardless of their symptomatology [8]. For contact tracing to effectively reduce the time infectious cases are in the community, thus reducing the reproductive number (R) of the virus, enough testing must be readily available and deliverable [9]. The need for reliable and fast diagnostics has only been exacerbated in the last year, as even after the SARS-CoV-2 vaccine roll-out, the pandemic has persisted and highly transmissible variants are arising [10]. As we approach a scenario of relative control of the transmission and reduced prevalence, highly accurate diagnostic assays become even more relevant. It has been measured that in a COVID-19 low-prevalence population, even highly sensitive and specific tests return a high number of false positives [11].

Even though rapid testing [12] and self-testing [13] have exploded during the pandemic, most tests for the detection of viral pathogens besides SARS-CoV-2 are performed in reference laboratories. Whilst tests themselves can be short, samples need to be shipped to the reference facility where they may be halted in a long queue for testing, causing an effective sample-to-answer time of days rather than minutes or hours. During the pandemic response, experiences in different mass screening set-ups, such as drive-through testing, have clearly exposed the importance of delivering test results to the patients before they leave the testing site. It has been proven that for a sample-to-answer time of 72 h, even several weeks of subsequent isolation and quarantine of the patient would have no effect on disease spread. To avoid this, tests need to not only be fast, but also be performed on-site, using sample-to-answer designs dependent only on modest equipment. Moreover, they must be able to process a high number of samples simultaneously [14,15].

**Figure 1:**
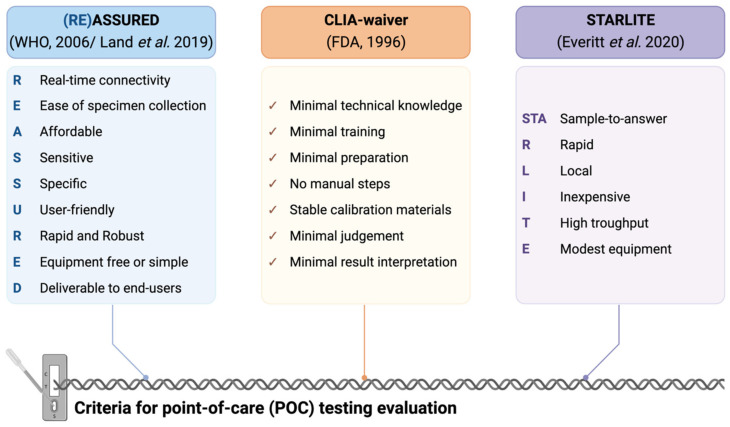
Requirements of the (RE)ASSURED [17,18], CLIA [19] and STARLITE [14] criteria for point-of-care diagnostics. Created with Biorender.com (accessed on 10 October 2022).

Under resource-limited conditions, each diagnostic test—antigen detection, antibody detection, or nucleic acid amplification tests (NAATs)—shows advantages and shortcomings in terms of specificity, sensitivity, accuracy, cost, etc. Specifically for SARS-CoV-2, accuracy and specificity is highest for NAATs, while antigen and antibody testing are faster and less costly [16]. To make sense of this, several criteria have been defined to assess point-of-care (POC) applicability of tests, detailed in Figure 1. The World Health Organization (WHO) relies on the ASSURED criteria [17]. Defined in 2004, recent proposals have been made to update it, standing now as the REASSURED criteria [18]. For the Food and Drug Administration of the United States (FDA), the standard is based on the Clinical Laboratory Improvement Amendment (CLIA), which builds upon the ASSURED criteria, expanding on the user-friendly term to include minimal training and no need for precise measurements, user interpretation or intervention [19]. Since the advent of the COVID-19 pandemic, other benchmarks such as STARLITE have been presented, focusing on pandemic response rather than technological dogmas [14].

In reference laboratories, where equipment and resources are available, NAATs are preferred over antigen tests due to their higher specificity and sensitivity, partly achieved by the amplification process that is lacking in antigen tests [20]. Currently, the gold-standard for diagnosis of viral infections is the reverse-transcriptase polymerase chain reaction (RT-PCR). For SARS-CoV-2 it has shown impressive sensitivity, detecting under 100 copies of the RNA target per mL (cp/mL) [21]. However, this technology is poorly adaptable to POC diagnostics mainly due to the temperature cycling requirements [22].

Since the early 1990s, various nucleic acid isothermal amplification assays have been proposed as a promising alternatives to PCR for POC testing [23]. In this review, we will focus on the adaptation of NAATs, both RT-PCR and isothermal nucleic acid amplification, for the POC diagnosis of COVID-19 and their role in the response to present and future outbreaks. We will define the advantages and disadvantages of different technologies, strategies to improve signal detection and read-out, as well as clinical validation using different specimens. We will also explore the combination of NAATs with CRISPR-based detection systems and the diagnostic challenge posed by SARS-CoV-2 variants. Lastly, we will discuss the impact that these technologies have had in the clinic as well as the future perspectives after the COVID-19 pandemic.

## 2. RT-PCR as a Point-of-Care Tool

The FDA, the WHO and the European Center for Disease Control (ECDC) agencies have different criteria to grant temporary approval to POC tests. Still, they all require at least 80% sensitivity (positive percentage agreement; PPA) and 97–98% specificity (negative percentage agreement; NPA) [24]. RT-PCR is a highly sensitive and robust technique; however, it is poorly adaptable to POC conditions. Nevertheless, it has some advantages over other alternatives such as isothermal amplification techniques. So far, RT-PCR has shown more success and consistency in the detection of SARS-CoV-2 RNA than isothermal amplification techniques. Additionally, the high-temperature requisites of RT-PCR, despite the increase in instrumentation cost and complexity, prevent to a great extent non-specific amplification, which is more common in isothermal amplification assays [25].

The FDA has approved under the Emergency Use Authorization (EUA) numerous NAATs. Some of them have even been authorized for home use, although no PCR-based assays have obtained this authorization yet. However, several RT-PCR tests have been granted the Clinical Laboratory Improvement Amendments (CLIA) Waiver, which allows their use in POC settings (see Table 1) [26]. A notable example is the Accula SARS-CoV-2 test. The assay provides a buffer for RNA extraction from nasopharyngeal (NP) swabs. The extracted RNA is then transferred to a cassette, which contains internal positive and negative controls. Then, RT-PCR is performed targeting the *N* gene of SARS-CoV-2 in the cassette and detection is achieved by lateral-flow, providing results in approximately 30 min. The assay claims a 95.8% PPA and a 100% NPA [27]. However, it has been subsequently tested in 100 samples and compared with reference laboratory diagnosis reaching an overall agreement between the assays of 84.0%. Compared to the reference diagnosis, the Accula SARS-CoV-2 test showed high negative agreement, but reduced positive agreement, especially for those samples with low viral load [28].

Other tests, such as the Xpert Xpress SARS-CoV-2/Flu/RSV test or the BioFire Respiratory Panel 2.1-EZ, have been designed for the simultaneous detection of various respiratory pathogens. With the Xpert Xpress SARS-CoV-2/Flu/RSV test, SARS-CoV-2, Influenza A and B and respiratory syncytial virus (RSV) are detected in a cartridge that can be ran in various GeneXpert devices, including those adapted to POC settings [29]. In a multicenter study a total of 319 NP swabs, the overall PPA for the SARS-CoV-2 was 98.7% and the NPA was 100%, with the detection of the other viruses showing complete agreement [30]. Additional studies using this kit have shown comparable results, both using upper respiratory tract specimens (NP swabs) [31] and low respiratory track specimens (bronchoalveolar lavages and tracheal aspirates) [32]. With the BioFire Respiratory Panel 2.1-EZ, a panel of ten human respiratory viruses and four human respiratory bacteria can be tested in a single run of the sample-to-answer device. Interestingly, when compared with the reference RT-qPCR, the device detected 48 out of the 49 SARS-CoV-2 positives. Considering 30% of them presented a cycle threshold (Ct > 30) in the reference RT-qPCR, thus a low viral load, it could be highly relevant in the clinic [33]. However, other authors have pointed out unreliable results for SARS-CoV-2 with this diagnostic kit and recommend to perform other RT-qPCR assays for result confirmation [34].

**Table 1:** RT-PCR tests for SARS-CoV-2 detection approved for use under a Clinical Laboratory Improvement Amendments (CLIA) Waiver.

Test	Targets	LoD (cp/mL) ^b^	Run	Specimen ^c^	n ^d^	PPA/NPA (%) ^e^	Devices	Samples per Run	Read-Out	Reference
**Monoplex**										
Xpert Xpress CoV-2 plus	N2, E and ORF1ab	70	30 min	NP swab NS swab	164 111	100/96.5 100/100	GeneXpert Dx GeneXpert Infinity	4 48/80	Real-time fluorescence	Cepheid [35]
Xpert Xpress SARS-CoV-2 test	N2 and E	125	30 min	NS swab	90	95.8/95.6	GeneXpert Dx GeneXpert Infinity	4 48/80	Real-time fluorescence	Cepheid [36,37]
cobas SARS-CoV-2	ORF1ab and N	12	20 min	NP swab	230	96.1/96.8	cobas^®^ 6800 cobas^®^ 8800	96 every 3 h 96 every 3 h	Real-time fluorescence	Roche [38]
MicroGEM Sal6830 SARS-CoV-2 Saliva Test	N and E	6.4 × 10^3^	30 min	Saliva	119	87.2/97.2	MicroGEM Sal6830 PoC PCR System	1	Real-time fluorescence	MicroGEM [39]
DASH SARS-CoV-2/S Test	N1 and N2	7.5 × 10^3^	16 min	NS swab	313	95.9/98.5	DASH Analyzer	1	Real-time fluorescence	Minute Molecular [40,41]
Visby Medical COVID-19 Point of Care Test	N1	1.1 × 10^3^	30 min	NP swab	95	100/95.3	Visby COVID-19 Device	1	Lateral-flow	Visby Medical [42,43]
Accula SARS-CoV-2 Test	N	150	30 min	NS swab	50	95.8/100	Accula Dock	1	Lateral-flow	Accula [44]
**Multiplex**										
Xpert Xpress CoV-2/Flu/RSV plus	N2, E and ORF1ab	138	30 min	NP swab	279	100/100	GeneXpert Dx GeneXpert Infinity	4 48/80	Real-time fluorescence	Cepheid [45]
Xpert Xpress SARS-CoV-2/Flu/RSV	N2 and E	131	30 min	NP swab	240	97.9/100	GeneXpert Dx GeneXpert Infinity	4 48/80	Real-time fluorescence	Cepheid [46]
cobas SARS-CoV-2 & Influenza A/B Nucleic Acid Test	ORF1ab and N	12	20 min	NP swab NS swab	935 930	95.2/99.6 96.4/99.5	Cobas^®^ Liat^®^ System	1	Real-time fluorescence	Roche [47]
BioFire Respiratory Panel 2.1-EZ (RP2.1-EZ) ^a^	S and M	500	45 min	NP swab	98	98/100	BioFire^®^ FilmArray^®^ System	1	Real-time fluorescence	BioFire [27]

^a^ Respiratory pathogens panel: Adenovirus, Human coronavirus (CoV) 229E, HKU1 CoV, NL63 CoV, OC43 CoV, SARS-CoV-2, Influenza A, Influenza B, Parainfluenza, RSV, *Bordetella parapertussis*, *Bordetella pertussis*, *Chlamydia pneumoniae*, *Mycoplasma pneumoniae.* ^b^ All concentration units are homogenized to copies per mL (cp/mL) from the original references for easier comparison. ^c^ NP swab: nasopharyngeal swab; NS: nasal swab. ^d^ n: sample size. ^e^ PPA: positive percentage agreement; NPA negative percentage agreement.

Outside the FDA CLIA waived technologies, other emerging RT-PCR POC tests provide valuable results. Noteworthy is the COVIDNudge platform [48], a lab-on-chip sample-to-answer device able to perform sample processing and RT-PCR outside the laboratory in under 90 min. It consists of two components, a DNA cartridge that provides sample-to-answer RT-PCR and a processing unit (Nudgebox). The DNA cartridge encompasses an amplification unit and a sample preparation unit, where RNA is extracted, and RT-PCR lyophilized reagents are added to the extracted RNA. Cartridges are placed in the Nudgebox processing unit, which allows a RT-PCR reaction to be run outside a laboratory setting. This POC platform was evaluated in 386 paired samples, showing an overall sensitivity of 94% compared to the laboratory-based test with an overall specificity of 100%.

Of note, exciting approaches to the POC diagnosis of SARS-CoV-2 have been made through the optimization of digitalPCR (dPCR). The main advantage of this technique is the absolute quantification capacity, accomplished via the partitioning of the reaction mix into many sub-reactions, avoiding the need for a standard curve or the use of reference genes. Additionally, it offers high sensitivity, precision, and resistance to inhibitors. However, most dPCR tests presented to date require a separate RNA extraction step, expensive instruments and trained personnel to operate the systems [25]. As an example, the FastPlex Triplex SARS-CoV-2 test is a digital droplet dPCR (ddPCR) able to detect three sequences (*ORF1ab*, *N* gene and *RNase P*). Both the droplet generation and the ddPCR are performed using a DropX-2000 Sample Prep Station. Validated in 168 samples, the POC test showed a 96.3% PPA and 96.7% NPA with the laboratory reference diagnostic [49]. A recent metanalysis confirmed that dPCR is more sensitive than both RT-PCR and RT-LAMP for the detection of SARS-CoV-2, although RT-LAMP shows the highest specificity [8]. Attending to those features, some authors suggest the technique could provide definitive results for borderline positive cases or suspected false-negative cases [50].

## 3. Loop-Mediated Isothermal Amplification (LAMP) as a Point-of-Care Tool

Since the 1990s, a wide arsenal of isothermal amplification tests has been developed with important differences amongst them in terms of probe and primer design, sequence targets, enzymatic activities, and reaction conditions. In this short period, an important body of literature has been presented in the application of isothermal techniques to detect SARS-CoV-2 [51].

Loop-mediated isothermal amplification (LAMP) amplifies DNA at a constant temperature (60–65 °C) making use of an strand-displacing polymerase, achieving high efficiency, specificity and velocity [52]. In less than one hour, LAMP amplifies DNA with an efficiency of up to 100 times superior to a conventional PCR [53]. The assay can be easily combined with reverse transcription in a one-step protocol (reverse transcription loop-mediated isothermal amplification (RT-LAMP)) by either adding a dedicated reverse transcriptase or a DNA polymerase with reverse transcriptase activity [22]. LAMP is the most used isothermal amplification technique for the development of POC tools [14]. Compared with other isothermal amplification techniques, LAMP presents some major advantages, including the use of a single enzyme, high specificity provided by the primer design, relatively low reaction time (<1 h) and high efficiency. However, it also shows some disadvantages, including relatively high reaction temperature (60–65 °C), complex primer design, not suited for cloning and difficult multiplexing [23].

Focusing on SARS-CoV-2 detection, RT-LAMP has been tested in a wide variety of specimens, including NP swabs, nasal (NS) swabs, saliva, sputum, endotracheal secretions and bronchoalveolar lavages (all of them with and without prior RNA purification) [54,55], urine [56], stool [57], sewage [58] and different surfaces of public spaces [59]. Rigorous studies comparing the performance of RT-LAMP are scarce [54,55] or lacking for some specimens, probably because they were available early on for RT-qPCR [60], which dissuaded researchers for further investigation.

In the past, one of the main limitations that has prevented LAMP from entering the “real-world” diagnostic practice has been the lack of clinical validation. This has completely shifted during the pandemic when various large-scale clinical studies have proven its applicability. A study was conducted to evaluate the performance of the OptiGene Ltd. SARS-CoV-2 RT-LAMP in 559 raw NP swabs and 86,760 raw saliva samples, as well as RNA extracted from over 12,000 NP swabs and saliva specimens. Analysis with purified RNA resulted in an 80.65% sensitivity in saliva and 96.06% in NP swabs, while raw samples yielded 70.35% sensitivity on swabs and, surprisingly, 84.62% in saliva. In all cases specificity was over 99.9% [54]. The use of extraction-free protocols in saliva has been further validated with other RT-LAMP assays, such as the SARS-CoV-2 Rapid Colorimetric LAMP Assay Kit, which was evaluated in pre-defined clinical cases and determined to be 97% sensitive and 100% specific. Then, it was tested in over 30,000 self-collected samples over a 10-month period, yielding results consistent with epidemiological data [61]. To further evaluate the potential POC application of the technique, another study assessed RT-LAMP performance in 4704 self-collected saliva samples. The assay, which used primer sets from the Color Genomics COVID-19 test in a 30 min run, was performed in two workplaces, two schools and an athletic program. Overall, the assay showed a 98.8% concordance with the reference RT-qPCR [62]. Lastly, a clinical evaluation of colorimetric RT-LAMP for SARS-CoV-2 detection was performed in four countries of sub-Saharan Africa and Italy. Comparing over 1000 NP swabs that underwent the same procedure in all countries, a sensitivity of 87% was obtained and a 98% specificity. The sensitivity was greatly affected by samples with very low viral load (RT-qPCR Ct > 35). Considering samples with a Ct < 35, sensitivity increased to 97% [63].

Thus, the available research suggests that, as a general rule, pharyngeal swabs provide more sensitive results than saliva when RNA purification is performed, while crude saliva samples provide better results than crude pharyngeal samples [54]. In terms of which type of pharyngeal sample is used, no significant differences in sensitivity or specificity have been found between NP and oropharyngeal swabs either for RT-PCR or RT-LAMP [8].

**Figure 2:**
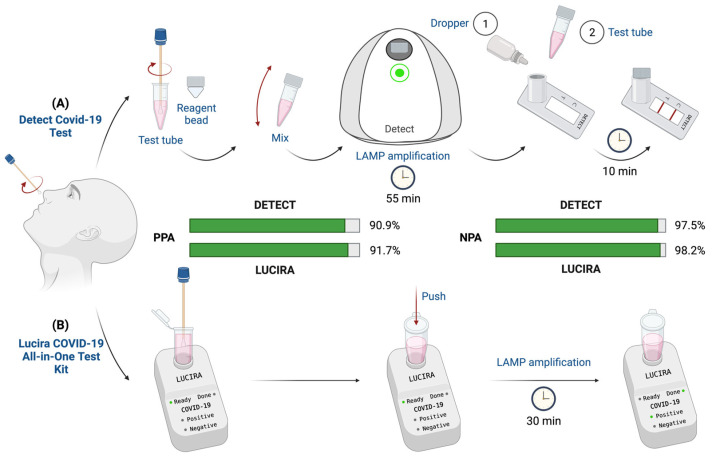
Workflows of the RT-LAMP assays approved by the Food and Drug Administration for home use. (**A**) Detect COVID-19 test workflow. A nasopharyngeal swab is self-collected by the patient and introduced in the test tube. Then, the test tube is closed with a cap containing reagent bead. Reagents are mixed with the sample and incubated for 55 min in the Detect hub to allow LAMP amplification. Finally, results are detected via lateral flow after another 10 min. (**B**) Lucira COVID-19 All-in-One test workflow. A nasal swab is self-collected by the patient and placed in the Lucira hub. The test tube is pushed down to initiate the LAMP amplification reaction. A positive or negative result is obtained after 30 min of incubation. Created with Biorender.com (accessed on 12 October 2022).

The emergency that has been caused by SARS-CoV-2 has propelled many RT-LAMP assays for SARS-CoV-2 to be granted the EUA by the FDA. Two of them, in contrast with the RT-PCR, have even been granted home use (Figure 2). The Detect COVID-19 test [64] uses RT-LAMP for the detection of the *ORF1ab* region of SARS-CoV-2 (Figure 2A). It provides a disposable test tube with collection buffer, which is used in combination with the Detect hub. When the tube is inserted in the Detect hub, amplification starts automatically and lasts for 55 min. After amplification, the tube is inserted into the reader to perform the lateral flow assay (10 min). The Detect app on a smartphone is utilized for the user result read-out. One of the main limitations of the assay is that the Detector hub needs to be set aside for about 65 min after plugging-in before running the test [65]. The other RT-LAMP assay authorized for home use is the Lucira All-in-one COVID-19 test [66], which uses self-collected NS swabs (Figure 2B). The RT-LAMP assay targets two non-overlapping regions of the *N* gene and gives results in less than 30 min. The read-out is based on a colorimetric change of a pH indicator during an amplification reaction. The swab needs to be inserted in the elution buffer and subsequently the sample is lysed at room temperature. The sample vial is then placed on the test unit and the eluant resolubilizes lyophilized reagents. The read-out is performed using optical and electronic elements in the test unit [67].

Another important shortcoming of RT-LAMP is the lack of an analogous alternatives to TaqMan probes in RT-qPCR, providing sequence-specific amplification detection. There are some other probe-base read-out systems that have been developed for LAMP, although they have proved to inhibit amplification to some extent and increase assay complexity [68]. Thus, extensive efforts have been made to improve readout strategies. A successful strategy for SARS-CoV-2 detection was presented utilizing a proofreading enzyme-mediated probe cleavage. This approach showed a detection limit of 100 cp in 50 min and was adaptable to multiplex detection exploiting different fluorophores [69].

Other read-out systems have approached POC via lateral flow assays, primarily with fluorescein isothiocyanate (FITC) or biotin-labeled primers. However, these readouts are not usually incorporated into the amplification and require additional handling steps [70]. The final read-out strategy are colorimetric approaches, that are less complex and easy to interpret, but they provide non-specific amplification signals and cannot detect multiple targets simultaneously [68]. In another study a pH indicator was used as a colorimetric readout, achieving 96.88% sensitivity on NP swabs, 94.03% on sputum and 93.33% on throat samples, while maintaining a 100% specificity [71].

The combination with portable devices or smartphone applications (mobile Health, mHealth) can simplify the read-out and achieve user-friendly detection. Notably, a RT-LAMP able to detect SARS-CoV-2 in under 20 min with a LoD of 10 cp/reaction was presented. The assay was integrated in a complementary metal oxide semiconductor (CMOS) ion-sensitive field-effect transistor (ISFET) POC platform, connected to a smartphone app to acquire and process the data. The device showed 91% sensitivity and 100% specificity in NP swab samples [72]. Another remarkable example is the Palm Germ-Radar (PaGeR), which combines colorimetric, fluorometric and lateral dipstick readouts, reaching a 1 cp/μL LoD in swab samples. However, it was tested only on a very limited number of samples [73]. Colorimetric approaches, although simple and user-friendly, can be affected by operator-bias. To avoid this issue, artificial intelligence algorithms have been coupled with RT-LAMP for the detection of SARS-CoV-2. Tested in around 200 COVID-19 patient samples, it showed 81.25% accuracy in the prediction of positive and negative results [74]. Our group has taken other approaches, developing the SMART-LAMP, a handheld device for real-time colorimetric isothermal assays. The amplification is detected by the real-time monitoring of colorimetric changes in the samples, provided by an inexpensive malachite green dye. Additionally, it was combined with ready-to-use reagents stabilized by simple desiccation (not lyophilization) and a smartphone application for management, control, result visualization and analysis. The device achieved an 88.3% sensitivity for SARS-CoV-2 detection in 80 NP swabs compared to RT-qPCR. Moreover, sensitivity was improved slightly to 95.0% if only RNA samples with an estimated viral load over 500 cp were considered [75].

## 4. Recombinase Polymerase Amplification (RPA) and Recombinase-Aided Amplification (RAA) as Point-of-Care Tools

Recombinase polymerase amplification (RPA) is a highly sensitive and specific isothermal amplification technique performed at a constant temperature of 37–42 °C. It relies on a recombinase protein uvsX from T4-like bacteriophages, which binds to a set of two primers (a set of two, analogous to PCR primers) in the presence of ATP and a crowding agent. When a homologous sequence to the primer is detected in the target, strand invasion takes place. To prevent ejection from the inserted primer, the displaced DNA strand is stabilized by single-stranded binding (SSB) proteins. Finally, the recombinase disassembles and a strand displacing polymerase (usually, *Bacillus subtilis* Pol 1) elongates the primer [76]. Similarly to LAMP, the assay can be easily combined with a prior retro-transcription, either in a two-step or one-step design [77]. The technique shows advantages over other molecular techniques, such as fast kinetics, low reaction temperature, a simple primer design and easy combination with probe-based detection. However, its low reaction temperature can also cause undesired amplification at room temperature or primer–primer interaction, even for those carefully designed [78].

Although RPA has proved to be a sensitive and specific technique as a stand-alone method, most assays developed for SARS-CoV-2 diagnosis couple it with another amplification or detection technology. It has been shown to be especially impactful when coupled with CRISPR-based detection, including CRISPR-Cas9 [79], CRISPR-Cas12a [80] and CRISPR-Cas13a [81] which will be discussed below. Additionally, an equivalent assay to RPA has been developed, utilizing a recombinase from *Escherichia coli* instead of one from phage T4, named recombinase-aided amplification (RAA), which allows for reaction performance at room temperature [82].

Clinical validation of the RPA or RAA techniques has shown to be far behind RT-PCR or RT-LAMP for either RPA or RAA. Three studies coming out of China have presented early evidence of RAA clinical validation. First, a multicenter study evaluated the application of RT-RAA for SARS-CoV-2 diagnosis in a rapid assay format taking only 15 min, performed at 39 °C with a portable device. Nevertheless, it used purified RNA. The study included 926 samples and showed 97.6% sensitivity and 97.9% specificity when compared to RT-qPCR [83]. Another report analyzed 506 samples with RT-RAA and claimed 100% specificity and sensitivity when compared to RT-qPCR [84]. Additionally, work was performed comparing the performance RT-RAA in 404 samples with two RT-PCR assays, targeting the *N* gene and ORF1ab, respectively, and a ddRT-PCR assay. The report showed that ddRT-PCR yielded the highest sensitivity, followed by RT-PCR targeting ORF1ab and RT-RAA, while RT-PCR detecting the *N* gene showed considerably lower sensitivity [85].

## 5. Other Isothermal Amplification Techniques as Point-of-Care Tools

The vast majority of isothermal NAATs for SARS-CoV-2 RNA detection are based on RT-LAMP and to a lesser extent RT-RPA or RT-RAA. Still, there are numerous isothermal techniques available for researchers and some have been employed for SARS-CoV-2 detection. A brief comparison of the isothermal NAATs addressed in this review is shown in Table 2.

A highly relevant isothermal amplification technology is the nicking endonuclease amplification reaction (NEAR), which is an extremely rapid molecular diagnostic technique used by Abbott to develop its own test against Influenza and SARS-CoV-2 [51] (Table 3). The amplification is achieved by the synergistic effect of a DNA polymerase and a nicking enzyme. Two nicking primers, containing a restriction enzyme site, a stabilizing region, and a target-binding region, are introduced to amplify a short single-stranded DNA (ssDNA) template. One of the primers hybridizes with the template making up a double-stranded intermediate. Then, the nicking enzyme breaks four bases downstream and the nicked primer can be extended again, displacing the strand downstream of the restriction site. The dissociated ssDNA product can hybridize with the other nicking primer and be extended by the polymerase. With the help of the nicking enzyme, the initial ssDNA template is generated, thus initiating another cycle of the amplification resulting in exponential amplification [89]. The NEAR-based assay developed by Abbott has shown the capacity to generate reliable results for SARS-CoV-2 detection in under 15 min [90,91].

Nucleic acid sequence-based amplification (NASBA) is based on the activity of the avian myeloblastosis virus reverse transcriptase (AMV RT), *Escherichia coli* RNase H and T7 RNA polymerase with two primers to amplify the target fragment more than 10^12^-fold in 90 min [92]. The technique has been applied in a two-stage testing strategy to SARS-CoV-2 detection. The platform, named INSIGHT [93], and designed for population-scale testing, combines POC diagnosis with next generation sequencing (NGS). The first stage gives results within 2 h, using either fluorescence detection or a lateral flow readout, while simultaneously incorporating sample-specific barcodes. The same reaction products from potentially hundreds of thousands of samples are then be pooled and used in a highly multiplexed sequencing-based assay in the second stage.

Exponential amplification reaction (EXPAR) is another isothermal NAAT which relies on a small amplicon (15 to 20 bases long), producing up to 10^8^-fold amplification of the target DNA in minutes. Briefly, a ssDNA fragment (the trigger) starts the EXPAR reaction by binding to the DNA template. Large quantities of short double-stranded DNA (dsDNA) sequences are then generated in an isothermal cycle involving a DNA polymerase to extend the sequence and a nicking endonuclease to cut it, while leaving the template unaltered [94]. This technology has been applied to SARS-CoV-2 detection, avoiding the need of a reverse transcription step to convert SARS-CoV-2 RNA into DNA. The assay involves the formation of an RNA/DNA heteroduplex, which is selectively cleaved, generating the trigger, that is then rapidly amplified using the exponential amplification reaction (EXPAR). This provides a single-step assay, whose results are detected via a fluorescence read-out, reaching a LoD of 7.25 RNA cp/µL of SARS-CoV-2 in under 10 min. Three-way comparison with both RT-qPCR and RT-LAMP showed that the EXPAR assay was faster while maintaining its sensitivity features [95].

Another well-known isothermal amplification is the rolling circle amplification (RCA). Using this technology, a paper-based SARS-CoV-2 assay based on net-like rolling circle amplification (NRCA) was developed, allowing for visual result detection in a few minutes [96]. A novel isothermal amplification method named CREA (circularization-RCA for extended amplicon) [97], has been used to copy long-amplicons of SARS-CoV-2. The assay utilizes sequence-specific recombination of Cre recombinase to generate circular intermediate templates for subsequent RCA reactions. The CREA method targeting the *spike* gene of SARS-CoV-2 was able to amplify a 2.9 kb target and up to 1.9 kb amplicons were able to produce in sufficient amount for cloning.

Preliminary studies have been performed using other isothermal amplification techniques. Reverse transcription helicase-dependent amplification (RT-HDA) has shown an impressive LoD of 3 cp/reaction to detect SARS-CoV-2, however has shown false positive results after 40 min of incubation [98]. A relatively new isothermal technique is the cross-priming amplification (CPA). The performance of the technique for the detection of SARS-CoV-2 has been compared to five different RT-PCR kits in a limited number of samples, yielding perfect accuracy [99]. A final isothermal amplification example is the transcription-mediated amplification (TMA) which has also been tested and compared with RT-qPCR for SARS-CoV-2 detection, showing over 99% sensitivity and specificity [100].

**Table 3:** Isothermal amplification tests for SARS-CoV-2 detection approved for use under a Clinical Laboratory Improvement Amendments (CLIA) Waiver.

Test	Use ^a^	Targets	LoD (cp/mL) ^c^	Run	Specimen ^d^	n ^e^	PPA/NPA (%) ^f^	Device	Samples per Run	Readout ^g^	Reference
**LAMP**											
Detect COVID-19 Test	POC Home	ORF1ab	800	55 min	NP swab	112	90.9/97.5	Detect Hub	1	Lateral flow App	Detect [64]
Lucira COVID-19 All-in-One Test Kit	POC Home	N	900	30 min	NS swab	404	91.7/98.2	Lucira Test Unit	1	Color change-LED detector	Lucira Health [66]
UOL COVID-19 Test	POC	N/D ^b^	2.6 × 10^3^	40 min	NP swab	207	87.7/100	UOL COVID-19 Instrument	1	Fluorescence App	Uh-Oh LABS [101]
DxLab COVID-19 Test	POC	M	6 × 10^4^	25 min	NS swab	139	86/100	DxHub Instrument	8	Fluorescence	DxLab [102]
**NEAR**											
ID NOW COVID-19	POC	ORF1ab	125	13 min	NP swab NS swab	207	94.5/99.3	ID NOW Intstrument	1	FLMB	Abbott [90,103]
ID NOW COVID-19 2.0	POC	ORF1ab	500	12 min	NP swab NS swab	438 430	92.5/98.4 94.0/98.6	ID NOW Intstrument	1	FLMB	Abbott [91]
**Qualitative Isothermal NAAT**											
Cue COVID-19 Test for Home and OTC Use	POC Home	N	1.3 × 10^3^	20 min	NS swab	273	97.4/99.1	Cue Instrument	1	App	Cue Health [104]
Talis One COVID-19 Test System	POC	ORF1ab N	500	27 min	NS swab	98	100/100	Talis One	1	Probe-specific fluorescence	Talis [105]

^a^ Test can be authorized either for home use (Home) or point-of-care use (POC). ^b^ N/D: Not disclosed. ^c^ All concentration units are homogenized to copies per mL (cp/mL) from the original references for easier comparison of the limit of detection (LoD). ^d^ NP swab: nasopharyngeal swab; NS: nasal swab. ^e^ n: sample size. ^f^ PPA: positive percentage agreement; NPA: negative percentage agreement. ^g^ App: a dedicated smartphone app presents the results to the patient; FLMB: fluorescently labeled molecular beacons.

## 6. Nucleic Acid Amplification Combined with CRISPR Diagnostics, a New Point-of-Care Approach

Clustered regularly interspaced short palindromic repeats (CRISPR)/CRISPR-associated (Cas) systems provide adaptive immunity against viruses and plasmids in bacteria and archaea. The silencing of invading nucleic acids is executed by ribonucleoprotein complexes preloaded with small, interfering CRISPR RNAs (crRNAs) that act as guides for targeting and degradation of foreign nucleic acids [106]. CRISPR–Cas systems can be divided, according to evolutionary data, into two classes and six types. The classes are categorized by the nature of the ribonucleoprotein effector complex: class 1 systems are characterized by a complex of multiple effector proteins, while class 2 systems encompass a single crRNA-binding protein. Among CRISPR systems, class 2 systems have predominantly been applied for diagnostics, as these systems are easier to recreate in vitro. They include enzymes with collateral activity, which serve as the backbone of many CRISPR-based diagnostic assays. Class 1 systems have also been engineered for diagnostics, although to a much lesser extent [107].

CRISPR-based detection of amplicons has been combined with most of the amplification technologies described here. Nevertheless, the most advanced CRISPR-based POC diagnostics for SARS-CoV-2 use either RT-LAMP or RT-RPA for amplification. Using RT-LAMP as the amplification technique is the SHERLOCK assay [108], which combines RT-LAMP with the thermoresistant Cas12b enzyme from *Alicyclobacillus acidiphilus*, allowing for a one-step assay. This assay, combined with a rapid RNA extraction method based on magnetic beads, showed applicability as a POC assay when evaluated using 402 NP samples, showing 93.1% sensitivity and 98.5% specificity. Other researchers have combined RT-LAMP with Cas12a achieving positive results in a 45 min one-pot assay [109] or even with new Cas13 variants known as miniature Cas13 (mCas13) [110].

In recent years, and intensely during the COVID-19 pandemic, the popularity of RPA techniques has experienced an important resurgence due to its combination with CRISPR diagnostics. Similar to RT-LAMP, SHERLOCK technology has been combined with RT-RPA. Here, two notable platforms are highlighted: minimally instrumented SHERLOCK (miSHERLOCK) and combinatorial array reactions for multiplexed evaluation of nucleic acids (CARMEN). miSHERLOCK [111] combines the SHERLOCK technology with a POC diagnostic platform that uses unprocessed saliva samples and performs all steps (extraction, amplification, and detection) in a sample-to-answer design in 1 h. Results can be evaluated with a dedicated smartphone app. Nevertheless, the assay has only been tested in a small set of specimens (*n* = 48). The development of the CARMEN platform has been particularly relevant [112], which allows for massively multiplexed detection of targets. In this platform, nanoliter droplets containing CRISPR-based detection reagents self-organize in a microwell array to pair with other droplets of RPA-amplified samples, theoretically testing each sample against each crRNA. The combination of CARMEN and Cas13 detection enables robust testing of more than 4500 crRNA and target pairs on a single array. With this platform, the simultaneous differentiation of 169 human-associated viruses has been achieved. Moreover, the new upgrade of the CARMEN platform (mCARMEN), which uses commercially available Fluidigm microfluidics and instrumentation, has been also recently applied to the diagnosis of SARS-CoV-2 [113]. The diagnostics method was tested with 525 NP swabs, simultaneously detecting SARS-CoV-2 and eight other respiratory viruses, achieving 93.3% PPA and 97.9% NPA compared with RT-qPCR.

Although the potential of this assay is enormous, it deviates from POC testing and rapid diagnostics, which have also been achieved combining RT-RPA and CRISPR diagnostics. A sample-to-answer 20 min assay based on RT-RPA and Cas12a was tested with 204 NP samples obtaining 94.2% sensitivity and perfect specificity [114]. The use of Cas12a in combination with RT-RPA has consistently showed LoD under 5 cp/reaction [115,116]. Besides Cas12 and Cas13, Cas9 has also been used in combination with RPA, although it lacks collateral cleavage activity in which the previous assays are based on for detection. Worth mentioning is an assay which simultaneously detects *E* and *ORF1ab* genes of SARS-CoV-2 in a single test. The result readout is made by a CRISPR/Cas9-mediated triple-line lateral flow assay (TL-LFA). It has shown a LoD of 100 cp/reaction. It was tested with 64 NP swab samples, showing 100% NPA and 97.14% PPA compared with RT-qPCR [79].

## 7. Point-of-Care SARS-CoV-2 Variant Diagnostic Challenge

The continuous appearance of new SARS-CoV-2 variants has posed a significant diagnostic challenge during the COVID-19 pandemic. NAATs are greatly affected by the mutations presented in the new variants due to their high specificity. Reports show SARS-CoV-2 mutations were most abundant, even in the early stages of the pandemic, on the targets of various *N* gene primers and probes used globally to diagnose COVID-19 by RT-qPCR [117]. Nevertheless, for POC tests, the problem has been tackled via different approaches. In the case of RT-LAMP, some assays were able to detect but not differentiate different variants. This was achieved either including specific primers for different variants, such Gamma, Zeta, Delta, B.1.1.374 and B.1.1.371, with a simple colorimetric assay [118] or selecting conserved regions to provide variant-resistant diagnostic assays [119]. Discrimination among variants has been achieved too through careful primer design combined with different technologies. Early on in the pandemic, through primer spatial separation in a microfluidic design, discrimination of the Alpha variant from the original virus was achieved [120]. The development of CRISPR-based diagnostics allowed for the simultaneous detection and discrimination of variants. RT-LAMP was combined with Cas12b in a single-pot assay (CRISPR-SPADE) and was applied to Alpha, Beta, Gamma, Delta and Omicron and validated with 208 clinical samples. A 96.7% accuracy of variant discrimination was obtained in a 10–30 min assay [121]. Moreover, a lyophilized version of the reagents was developed and combined with a portable multiplexing device capable of interpreting two fluorescence signals for POC applications. In the case of RT-RPA diagnostics, combined with CRISPR diagnostics, the mCARMEN platform, described above, was able to discriminate between Alpha, Beta, Gamma, Delta, Epsilon and Omicron variants and to detect specific single nucleotide polymorphisms (SNPs) present in one or more of the variants. The assay was validated in 2088 specimens, with almost perfect concordance to sequencing results [113]. A different approach was the combination of RT-LAMP with a bioluminescence assay (LAMP-BART); in this case, through a peptide–nucleic acid probe the correct discrimination of the L452R spike mutation was possible [122].

## 8. Perspectives

The lack of affordable and simple molecular diagnostics for infectious diseases, particularly in developing countries, has been a long standing bottleneck in the improvement of health systems [123]. Developing countries have a disproportionately large share of the global burden of disease, while also presenting an unreasonably low portion of global health-care resources [124]. The lack of access to accurate, high-quality and affordable diagnostic tests can lead to overtreatment, undertreatment, lack of treatment, unnecessary or even harmful treatment, thus also obscuring epidemiological data [125]. In fact, it is predicted that COVID-19 data reported to the WHO, although overwhelming, is most likely only a fraction of the total cases and deaths caused by SARS-CoV-2 [126].

The COVID-19 pandemic has generated two contrasting effects when it comes to health systems in developing countries. On the one hand, the crisis has imposed an additional burden to already fragile health systems, aggravated by a lack of quality data to correctly manage the disease, a lack of research funding to inform policy-making and a lack of a well-designed agenda to set research priorities [127]. On the other hand, the unprecedented research effort during the pandemic has resulted in new scientific, technological and infrastructural developments that could have a significant impact on the life quality of the population of those regions. At this time, it remains difficult to assess if the overall impact will be beneficial or detrimental in coming years.

Focusing on the molecular diagnostic landscape alone, the sheer testing volume, which in April 2022 exceeded 3 thousand million tests worldwide [24], has never been seen before. Additionally, the array of diagnostic options available, over one thousand NAATs or antigen-based tests are commercially available worldwide, has never been this high. Furthermore, rapid lateral-flow tests, RT-qPCRs or antigen tests, are now covered daily in the media and discussed by politicians and the public and are even available for home self-use, at least in high-income countries. Still, this urgent need for diagnostic capacity and testing has further broadened the gap between high and low-middle income countries, where the building capacity is needed most [125]. Particularly in the case of NAATs, which for the most part require extensive infrastructure, are labor intensive, require strict and efficient transport chains and are, therefore, expensive and further contributes to unfair testing access [128].

The role of isothermal NAATs in the COVID-19 pandemic response has been of the utmost importance, allowing for the use of NAATs even as at-home tests [67,70,104]. Still, they have not displaced RT-qPCRs as the primary choice for performing SARS-CoV-2 detection. The reasons behind this may include the difficulty to perform fair comparisons amongst NAATs [129], the robustness and widespread use of RT-qPCR [130] or the perceived immaturity of isothermal NAAT technologies in the market [131]. Type of specimen, sample collection, extraction protocol, amplification mechanism, read-out strategy or the analysis platform are only a few of the variables that are in play when comparing NAATs. Additionally, the semi-quantitative nature of RT-qPCR-based protocols, grounded mainly on Ct values rather than exact viral loads, is also a factor that contributes to the comparison impairment of test performances [129]. However, institutions are starting to open up to new technologies, as shown by the approval of numerous isothermal NAATs both for laboratory and outside the laboratory tests. The continuous development of supportive technology, including microfluidics, mHealth or nanotechnology, will benefit isothermal technologies in terms of sensitivity, specificity, robustness or adaptation to the detection of rising virus variants. Moreover, some of the limitations classically showed by isothermal amplification assays, such as the limited multiplexing capacity, have been mostly resolved [113].

The question that remains is if (and how) the leap in the molecular diagnostic field during the COVID-19 pandemic will shape future diagnostic guidelines for other infectious diseases. In addition, if (and how) the good intentions manifested by politicians and international agencies will turn into actionable steps to tackle diagnostic limitations. Populations in resource-limited countries are in desperate need for diagnostic interventions such as the ones experience during the SARS-CoV-2 emergence. A wide range of diseases including malaria, tuberculosis (TB), human immunodeficiency virus (HIV), hepatitis B, syphilis or neglected tropical diseases (NTDs) would greatly benefit from more accurate and easy-to-use diagnostic tools. It is calculated that over 35% of cases of TB are missed every year due to the lack of affordable diagnostics [132] and between 35% to 62% for other infections such as HIV, hepatitis B or syphilis in pregnant women [133]. On the bright side, the technology developed for the COVID-19 response is available and it has even shown optimistic preliminary results for other infections. For example, SHERLOCK-based diagnostics have now been applied to malaria detection [134] with great success even without nucleic acid extraction [135]. Moreover, some isothermal technologies are now recognized as essential in vitro diagnostics by the WHO, specifically TB-LAMP for tuberculosis diagnosis [136]. Scientist in the field have been pushing for years for isothermal NAATs to be included into our POC diagnostic arsenal. After the COVID-19, we cannot miss the chance to implement them and deliver them to the populations that needed them the most.

## 9. Conclusions

The COVID-19 pandemic has led to an unparalleled development and application of new diagnostic tests at the largest of scales. The adaptation of RT-PCR assays to point-of-care settings and the approval of numerous isothermal NAATs for home and point-of-care use has proven the maturity and robustness of these technologies in their “real-world” applications. Counterintuitively, this building capacity has further broadened the gap in diagnostic access between high and low-middle income countries. In all, SARS-CoV-2 response has led to unequivocal scientific advancement, which could translate into significant upgrades in the management and control of many infectious diseases and consequently, the improvement of the overall health of many people around the world. But to achieve this, pharmaceuticals, politicians and international agencies must take actionable steps to apply them and maintain research investment in the diagnostic field after the pandemic is under control.

## Figures and Tables

**Table 2:** Comparison of the main characteristics of isothermal nucleic acid amplification techniques used for SARS-CoV-2 detection. Data from [86,87,88].

Technique	Acronym	Temp. (°C)	Time (min)	Efficiency ^a^
Loop-mediated isothermal amplification	LAMP	60–65	30–60	10^9^
Recombinase polymerase amplification	RPA	37–42	30–90	10^7^–10^8^
Recombinase aided amplification	RAA	39	30–90	10^7^–10^8^
Nicking endonuclease amplification reaction	NEAR	60	15–30	10^9^
Nucleic acid sequence-based amplification	NASBA	41	90–120	10^6^–10^9^
Exponential amplification reaction	EXPAR	60	<30	10^6^–10^8^
Rolling circle amplification	RCA	60	90	10^3^
Helicase dependent amplification	HDA	37–65	30–120	10^6^
Transcription mediated amplification	TMA	37	60–120	10^6^

^a^ Accumulation of nucleic acid products at the end of the reaction, measured as fold amplification.
